# Analysis of Interaction Between Interfacial Structure and Fibrinogen at Blood-Compatible Polymer/Water Interface

**DOI:** 10.3389/fchem.2018.00542

**Published:** 2018-11-08

**Authors:** Tomoya Ueda, Daiki Murakami, Masaru Tanaka

**Affiliations:** ^1^Graduate School of Engineering, Kyushu University, Fukuoka, Japan; ^2^Institute for Materials Chemistry and Engineering, Kyushu University, Fukuoka, Japan; ^3^Frontier Center for Organic System Innovations, Yamagata University, Yamagata, Japan

**Keywords:** blood-compatibility, poly(2-methoxyethyl acrylate), intermediate water, interfacial structure, atomic force microscopy, fibrinogen

## Abstract

The correlation between the interfacial structure and protein adsorption at a polymer/water interface was investigated. Poly(2-methoxyethyl acrylate)(PMEA), which is one of the best blood compatible polymers available, was employed. Nanometer-scale structures generated through the phase separation of polymer and water were observed at the PMEA/phosphate buffered saline interface. The interaction between the interfacial structures and fibrinogen (FNG) was measured using atomic force microscopy. Attraction was observed in the polymer-rich domains as well as in the non-blood compatible polymer. In contrast, no attractive interactions were observed, and only a repulsion occurred in the water-rich domains. The non-adsorption of FNG into the water rich domains was also clarified through topographic and phase image analyses. Furthermore, the FNG molecules adsorbed on the surface of PMEA were easily desorbed, even in the polymer-rich domains. Water molecules in the water-rich domains are anticipated to be the dominant factor in preventing FNG adsorption and thrombogenesis on a PMEA interface.

## Introduction

When blood comes into contact with a foreign material, various biological defense systems, such as the complement, blood coagulation, and inflammation systems are promptly activated (Gorbet et al., 2004; Anderson et al., 2008; Sperling et al., 2009; Arima et al., 2010). Thus, medical devices such as artificial blood vessels, catheters, and stents require high antithrombogenicity to suppress thrombus formation on the material surface. These biological reactions are triggered by the adsorption of proteins on the material surface in a biological environment (Chen et al., 2008). Hence, research evaluating the state of adsorbed proteins, e.g., the amount, composition, distribution, conformation, and orientation, have been conducted. Furthermore, significant effort has been paid to their control using interfacial parameters such as the wettability (Herrwerth et al., 2003), surface potential (Pasche et al., 2005), topography (Okano et al., 1981), molecular mobility (Nagasaki, 2011), and hydration (Chen et al., 2010; Vogler, 2012) of the material surface.

Poly(2-methoxyethyl acrylate) (PMEA) is a synthetic polymer with excellent blood compatibility (Tanaka et al., 2000b). Notable characteristics of PMEA include a unique interaction to water molecules, which play a dominant role in a biological environment and are detected using differential scanning calorimetry (DSC) (Hatakeyama and Hatakeyama, 1998), infrared spectroscopy (Morita et al., 2007), nuclear magnetic resonance (Miwa et al., 2009), and other approaches. Using DSC measurements, Tanaka et al. classified water molecules interacting with PMEA into three types: free water, freezing-bound water (intermediate water), and non-freezing water (Tanaka et al., 2000a). It has been clarified that the adsorption and conformational alteration of proteins on PMEA-type polymers are inhibited when the proportion of intermediate water among the three types of water increases (Sato et al., 2015). Furthermore, intermediate water is also observed in the blood-compatible synthetic polymers, polyethylene glycol (PEG) (Hatakeyma et al., 2007) and poly(2-methacryloyloxyethyl phosphorylcholine) (PMPC) (Hatakeyama et al., 2010), and in biopolymers such as proteins (Kawai et al., 2006) and polysaccharides (Hatakeyama and Hatakeyama, 1998).

Our group recently conducted an investigation into the interfaces between PMEA analogs and water or phosphate-buffered saline (PBS) using atomic force microscopy (AFM) (Murakami et al., 2016). For blood-compatible polymers containing intermediate water, nanometer-scale protrusions spontaneously appeared at the interface. In contrast, for non-blood compatible polymers with less intermediate water, irregular large aggregates were observed. The interfacial structure formation is thought to be caused by a phase separation of polymer and water in the interface region. PMEA is known to be miscible with water in higher compositions in the interfacial region than in the bulk phase (Hirata et al., 2015). The phase separation occurs to reduce the free energy at the interface further. Because the mobility of polymer chains at the interface is restricted due to the entanglement of molecular chains in the bulk region, the phase separation occurs in the nanometer scale. Thus, polymer-rich domains (composed of major polymer component and poor water) and water-rich domains (composed of major water component and poor polymer) form at the interface. Because the tiny density of the polymer chains in a water-rich region cannot be detected using AFM, an uneven topographic image is obtained. Stiffness of each domain is several MPa by AFM. This value is obviously less than the stiffness of the substrate, indicating that the surface unevenness at PMEA/PBS is not generated by a dewetting phenomenon of polymer on the substrate. The relationship between the interfacial structure in a thermosensitive biocompatible polymer and the protein adsorption and cell adhesion was investigated by Murakami et al. (2018). As the region with polymer-rich domains at the interface decreases (in which the water-rich domains increase) with a change in temperature, the amounts of adsorbed fibrinogen (FNG) and platelet adhesion decrease. This result indicates that FNG, which is a scaffold for platelet adhesion, is selectively adsorbed into the polymer-rich domains. However, there have been no studies directly and quantitatively analyzing the interaction between the interfacial structures and proteins.

In this work, we investigated the relationship between the interfacial structures and FNG adsorption using AFM. FNG is one of the main components in plasma, and has an important role as a scaffold in the thrombus formation. Therefore, the amount and state of FNG at an interface are very important factors. To elucidate the mechanism of FNG adsorption, the interaction between FNG and each domain at an interface was investigated through force curve measurements using a cantilever covered with FNG. In addition, the distribution of adsorbed FNGs was investigated on the interface of the PMEA in an FNG-PBS solution, as well as the subsequently rinsed and dried PMEA surface.

## Materials and methods

### Materials

Fibrinogen from human plasma (FNG) was purchased from Sigma-Aldrich, USA. Human whole blood used for an enzyme-linked immunosorbent assay (ELISA) was obtained from Tennessee Blood Services, USA. A micro BCA protein assay kit (Thermo Fisher Scientific, USA), Blocking-One (Nacalai Tesque, Japan), antifibrinogen γ′ antibody (Merck Millipore, Germany), peroxidase-conjugated anti-mouse IgG Ab (Bio-Rad Laboratories, USA), and a 2,2′-azinobis(3-ethyl-benzothiazoline-6-sulfonic acid ammonium salt) (ABTS) substrate (Roche Diagnostics K. K., Japan) were used for the FNG adsorption tests.

### Sample preparation and characterization

PMEA (*M*_n_ = 31 kg/mol, *M*_w_/*M*_n_ = 3.33) and poly(butyl acrylate) (PBA) (*M*_n_ = 85 kg/mol, *M*_w_/*M*_n_ = 1.47) were synthesized by free radical polymerization, as described in a previous report (Tanaka et al., 2000b; Sato et al., 2015). The chemical structure is shown in Supplementary Figure 1. Polymer solutions (0.2% w/v polymer/solvent) were prepared by dissolving PMEA in methanol and PBA in ethanol. Before the film preparation, polyethylene terephthalate (PET) substrates (φ = 14 mm, thickness = 125 μm) were rinsed in ethanol and dried at room temperature for 1 h. Then, the polymer solution (40 μL) was spin-coated twice on PET using a Mikasa Spin Coater MS-A100, as previously reported (Kobayashi et al., 2017). The static contact angle of the sessile water droplet (purified using PURELAB Option, ELGA Labwater) on the polymer films was measured using a DropMaster DMo-501SA (KYOWA) (Supplementary Table 1). Polymer/PBS interfaces at 37°C were observed using AFM (Cypher, Oxford Inst., Inc.) with an environmental cell. A cantilever of a pyramidal silicon nitride tip, with a spring constant of 0.57 N/m and a resonance frequency of 73 kHz (in air) (TR800PSA, Olympus, Co.), was used.

### Evaluation of FNG adsorption and conformational alteration using micro BCA assay and elisa

The amount of adsorbed FNG was evaluated using a micro BCA assay. Polymer solutions of 11.2 μL (0.2% w/v) were cast onto 96-well polypropylene (PP) plates and dried over a 3 day period at room temperature. An FNG solution of 50 μL (1 mg/mL in PBS) was added to each well and incubated for 10 min at 37°C. After incubation, each well was rinsed seven times with PBS. The adsorbed FNG was then extracted by incubating with a solution of 5% sodium dodecyl sulfate and 0.1 N sodium hydrate (NaOH) for 120 min at 37°C. The absorbance at 570 nm was measured using the BCA kit and a plate reader (BIO-RAD). The standard bovine serum albumin was used to determine the amount of adsorbed FNG.

The degree of conformational change of adsorbed FNG on the polymer interface was evaluated using ELISA. The platelet-poor plasma (PPP) obtained by centrifuging whole human blood was added to each well and incubated for 10 min at 37°C. The conformational change in FNG was evaluated using an antifibrinogen γ′ antibody (Ab) as a primary Ab, and peroxidase-conjugated antimouse IgG Ab as a secondary Ab. The secondary Ab reacted with the ABTS solution (1 mg/mL was detected by measuring the absorbance at 405 nm using a plate reader).

### Measurement of interaction between FNG and polymer interface

The interaction between the FNG and polymer interfaces was measured using AFM (Cypher, Oxford Inst. Inc.). FNG was coated onto the surface of a cantilever (TR800PSA, Olympus, Co.) through physical adsorption from the FNG solution (1.0 mg/mL, in PBS) for 60 min at room temperature. The FNG-coated cantilever was rinsed with PBS and used without drying. The spring constant of the cantilever was determined by fitting the power spectrum of its Brownian motion (Hutter and Bechhoefer, 1993). Force measurements were conducted with approaching and retracting rate of 500 nm/s in PBS at 37°C, at 64 different points in the interface. The image of height mapping was also obtained to help the position control of cantilever on polymer-rich and water-rich domains.

### Direct observation of adsorbed FNG on polymer surface

Topography and phase images of the PMEA interfaces after FNG adsorption were obtained using AFM (tapping scan mode, cantilever: TR800PSA). The distribution of adsorbed FNG was observed in the FNG-PBS solution (1.0 mg/mL) at 37°C without drying the surface after an incubation period of 10 min. The polymer film was then rinsed three times with PBS and deionized water. After drying in a desiccator for 1 day, the sample surface was observed again in air.

## Results and discussion

### Adsorption proteins and denaturation behavior on polymer substrates

**Figure 1:**
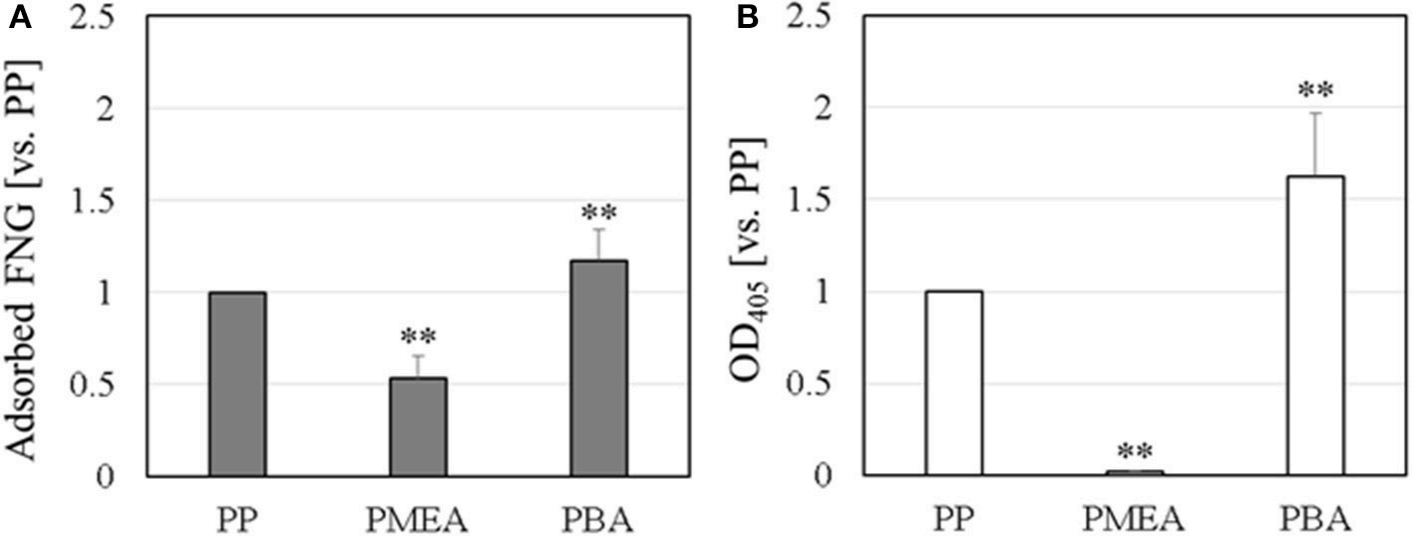
FNG adsorption behavior on polymer surface: **(A)** amount of adsorbed FNG, and **(B)** ELISA of the γ chain exposure in the adsorbed FNG. These data were normalized using PP and represent the means ± *SD* (*n* = 5), where ^**^p < 0.05 vs. PP.

**Figure 2:**
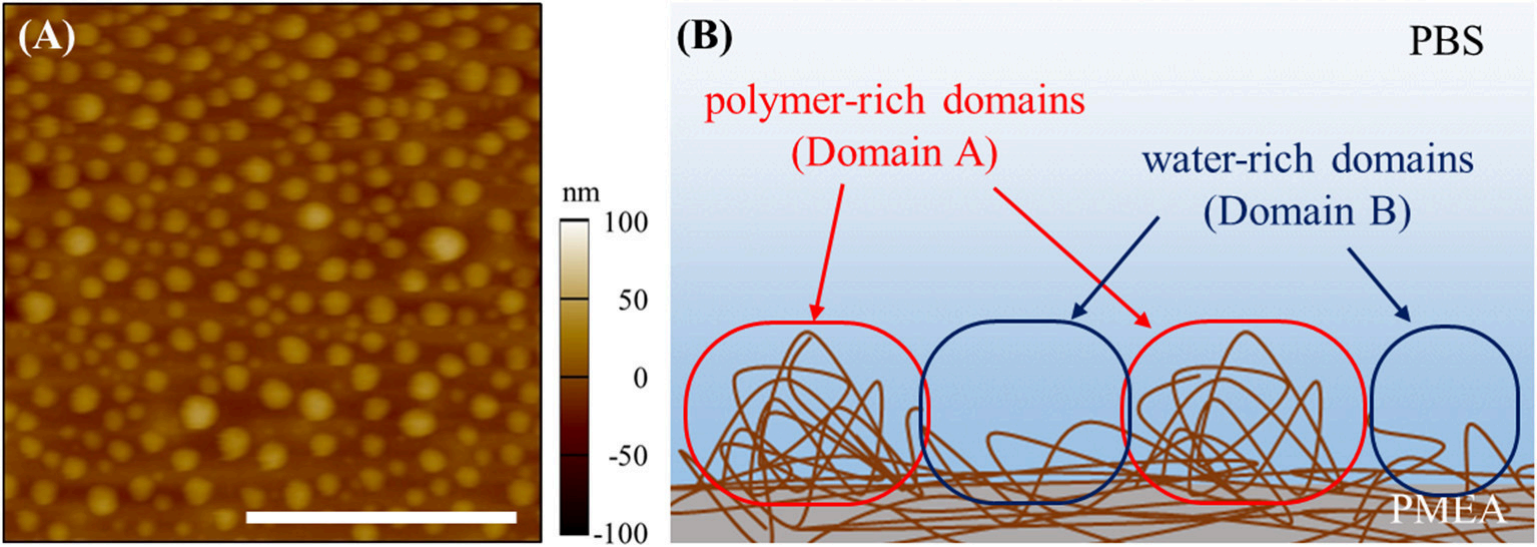
Nano-structures of PMEA/PBS interface: **(A)** topography of AFM image and **(B)** schematic image of nano-structures. The scale bar indicates 1,000 nm.

The results of the micro BCA assay are shown in Figure 1A. The amount of adsorbed FNG obtained on the polymer interfaces are normalized based on the amount of PP. For the PMEA, the amount of adsorbed FNG is suppressed more than the amounts of PP and PBA. At the PMEA/PBS interface, the polymer-rich domains (hereafter, Domain A, please see Figure 2) of the nano-structures are small and their distribution is relatively regular as compared to that of the PBA (for the features of the interfacial structures, please see Supplementary Figure 1). Recently, Murakami et al. reported that FNG molecules adsorb locally into Domain A or at the boundary parts between Domain A and the water-rich domains (Domain B) at a PMEA/PBS interface (Murakami et al., 2016). Hence, PMEA, which exhibits a small Domain A, consistently shows a low adsorption of FNG. Subsequently, we evaluated the exposure of the γ chain of adsorbed FNG, which works as a platelet activation site on the polymer surfaces, using ELISA. The results obtained are shown in Figure 1B as values relative to the PP. The degree of exposure of the γ chain in FNG was lower in the PMEA, and higher in the PBA. In conclusion, the adsorbed FNG on the PMEA is presumed to cause little conformational change, and is close to the natural state. According to the circular dichroism spectroscopy previously reported by Tanaka et al., FNGs adsorbed on the PMEA surface are known to retain their approximately native state (Tanaka et al., 2002), which is in good agreement with our present results. It was reported that the aggregation of multiple proteins promotes their degeneration (Weisel, 1986; Meadows et al., 2003; Yeromonahos et al., 2010). In the case of the PMEA, the size of Domain A (approximately 10–50 nm in height and 100 nm in diameter) is comparable to the molecular size of an FNG molecule (length of approximately 50 nm) (Hall and Slayter, 1959), and one or more FNG molecules can adsorb into a domain. This seems to prevent the denaturation of FNG through aggregation. In contrast, the amount and degree of γ chain exposure of adsorbed FNG on PBA were higher compared with the PP. It is thought that no suppression of FNG adsorption occurred and that the aggregation among the proteins enhanced the degeneration on the PBA.

### Interaction between interfacial structures and FNG

**Figure 3:**
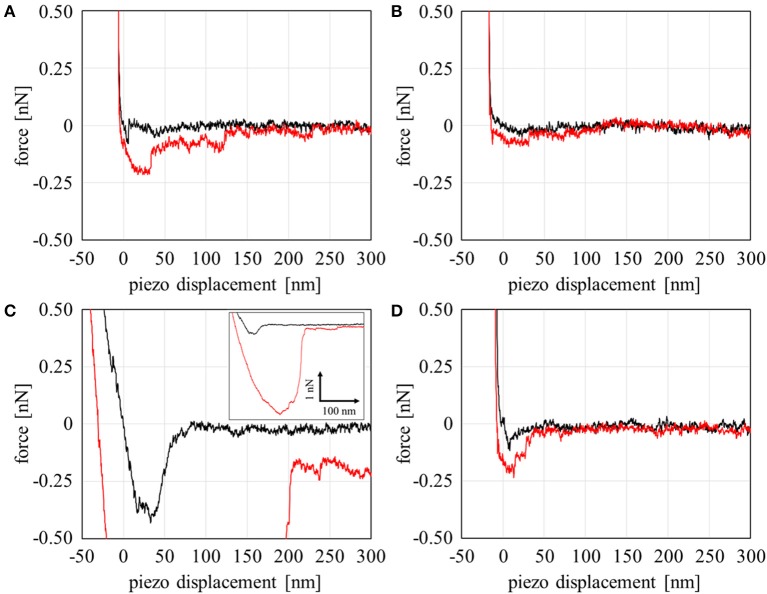
Force curve profiles on the approaching (black line) and retracting (red line) processes with a combination of the polymers and fibrinogen in PBS: **(A)** PMEA Domain A, **(B)** PMEA Domain B, **(C)** PBA Domain A, and **(D)** PBA domain B.

Our group recently reported that FNG molecules selectively adsorb into Domain A, which is spontaneously formed at the PMEA/water interfaces (Murakami et al., 2016). This behavior may be closely related to the low adsorption and denaturation of FNG on the PMEA, as mentioned above. To analyze the FNG adsorption more directly and quantitatively, the interaction between FNG and the nano-structures at polymer/water interfaces was examined through force curve measurements. Figure 3 shows the force-curve profile obtained in the approaching and retracting processes. The horizontal axis shows the z-position of the piezo stage, and the vertical axis shows the detected force. If the force is positive, a repulsive interaction occurs, and if the force is negative, an attractive interaction occurs. In this experiment, the detectable minimum force value was estimated to be 30 pN due to the thermal fluctuation of cantilever (see Supplementary Figure 3 for the detail). So the forces detected over 30 pN were regarded as substantial repulsive or attractive interactions. Here, force curves in approaching and retracting processes showed similar tendency. The attractive interaction (adhesion force) between a polymer and FNG was observed in Domain A of the PMEA (Figure 3A). In contrast, no attractive interaction was detected, and only the repulsion occurred in Domain B (Figure 3B). For the PBA, a strong adhesion could be observed in Domain A, and a detectable attractive force was presented even in Domain B, unlike with the PMEA (Figures 3C,D). This phenomenon is attributable to the composition of the polymer and water at each polymer domain. The most notable point is that only repulsion was observed in Domain B of the PMEA. A similar repulsive force was reported by Hayashi et al. (2007, 2012), who measured the interaction force between various functional groups on a self-assembled monolayer (SAM). A strong repulsive interaction within a separation range of 4–6 nm is observed when two or more ethylene glycol (EG) units are included in a molecule. The authors concluded that this repulsive interaction originates from the structured interfacial water layers on the SAM surfaces. A repulsive interaction from hydrated layers at the interface has also been demonstrated through molecular dynamics simulations (Pertsin and Grunze, 2011, 2014). We also expected that the origin of the repulsive interaction between Domain B of the PMEA and the FNG may be a physical barrier of hydrated water molecules. In addition, Tanaka's group reported (Tanaka and Mochizuki, 2004; Sato et al., 2015; Kobayashi et al., 2017) that the FNG adsorption on polymer films is suppressed as the amount of intermediate water in the polymers increases. Thus, it is predicted that for Domain B, i.e., the water-rich domain of the PMEA can retain a large amount of intermediate water at the interface, and prevent the adsorption of FNG.

**Figure 4:**
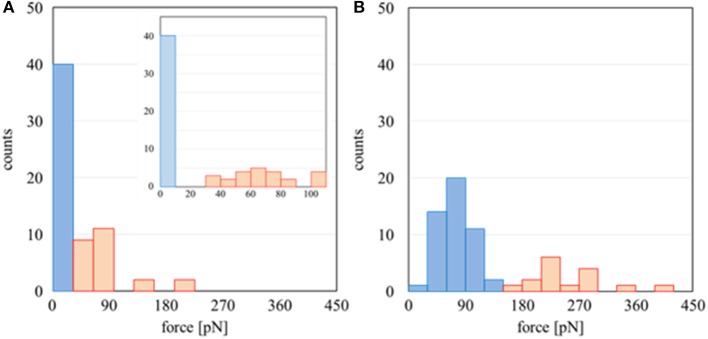
Histogram of adhesion forces of **(A)** PMEA/FNG and **(B)** PBA/FNG. The red and blue bars indicate the force curve measurement carried out on Domains A and B, respectively.

The results above were analyzed based on a histogram of the adhesion force obtained at 64 different points at the interface (including both Domains A and B). Herein, the adhesion forces were evaluated as the difference between the minimum and average values of the baseline plateau in a force curve. Herein, the force curves in approaching process were used for the analysis, since strong adhesive properties of PMEA and PBA may disturb the accurate evaluation of adhesion force in retracting process. Figure 4A is a histogram for PMEA, and Figure 4B is a histogram for PBA. First, regarding the interaction between the PMEA and FNG (Figure 4A), the adhesion on Domain B was almost zero or quite weak, less than detection limit. Even for Domain A, the adhesion was within 30–90 pN. For PBA (Figure 4B), on the other hand, it was found that the adhesion force was strong, particularly in Domain A, and converged at around 210 pN. Furthermore, it should be noted that the FNG was somewhat adsorbed even in Domain B. Because this result differs greatly from the attractive force between the PET as a substrate and FNG, the reason for the strong adhesion is not the influence of PET (please see Supplementary Figure 4). Weak hydration around the PBA chains may be insufficient in preventing fibrinogen adsorption even in Domain B. These results agree well with the adsorption behavior of FNG on PMEA and PBA, as macroscopically evaluated in Figure 2.

### Distribution of adsorbed FNG on PMEA surface

**Figure 5:**
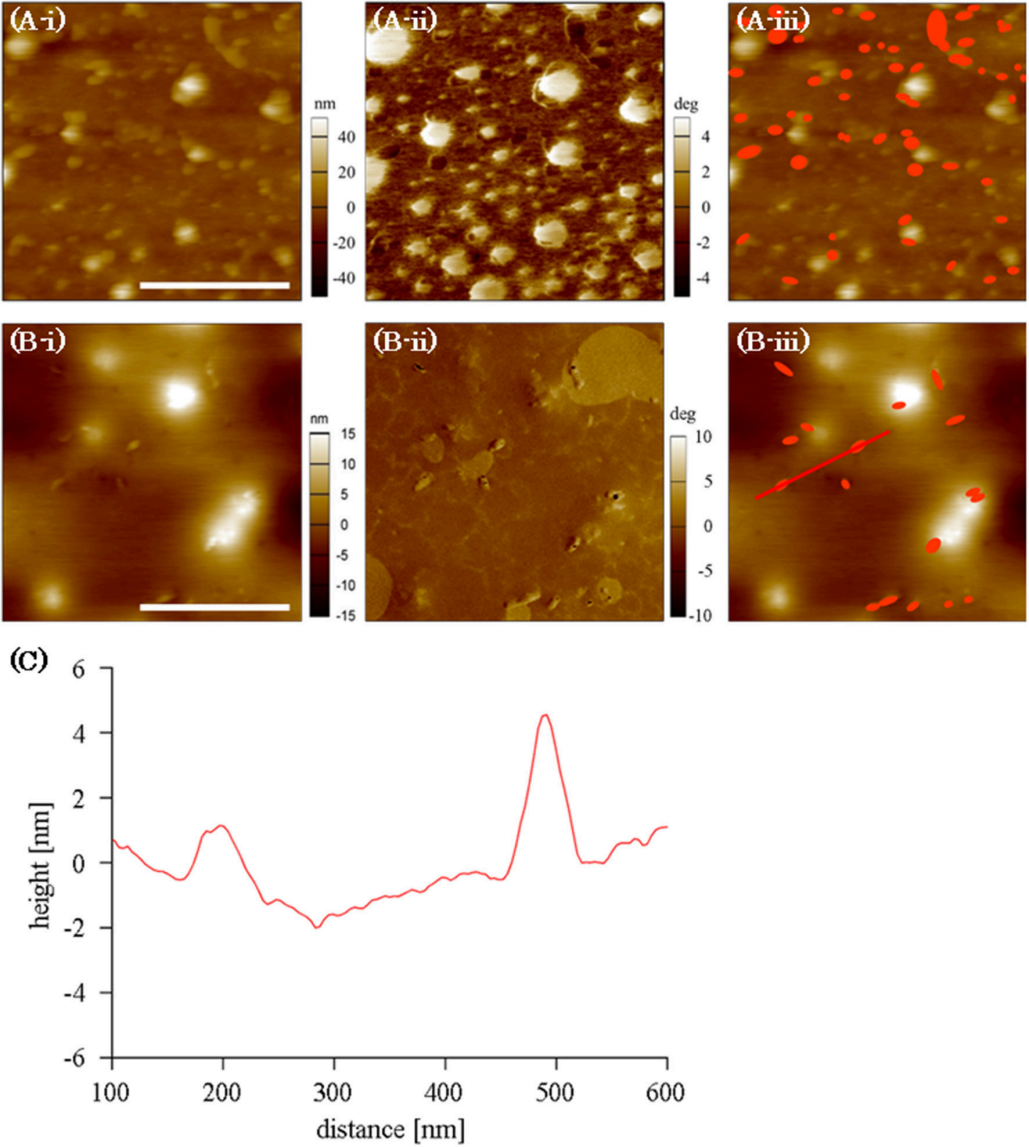
AFM images of FNG at PMEA interface: **(A)** in an FNG-PBS solution, **(B)** after rinsed and dried. **(Bi–Biii)** Indicate topographic images, phase images, and topographic images with the locations of the FNG molecules shown in red, respectively (scale bar indicates 500 nm). **(C)** The cross-section along the line shown in B-iii.

The distribution of adsorbed FNG molecules at the PMEA/PBS interface was observed using AFM (Figure 5A). Because the adsorbed FNG molecules are not clear in the topographic image (Figure 5Ai) owing to the nano-structures at the PMEA interface, the difference in phase value (Figure 5Aii) was used to recognize the location of the FNG molecules. The phase image at the PMEA/PBS interface without FNG is generally classified into two parts with different values, i.e., Domains A and B, as shown in Supplementary Figure 2. In the FNG solution, however, we confirmed the presence of some granular regions that show a lower phase value than the above two parts (Figure 5Aii). After the PMEA film with adsorbed FNG was rinsed and dried, the nano-structures almost disappeared because they were formed through a phase separation of the polymer and water at the interface, as previously reported (Murakami et al., 2016). In this case, components the same size as the granular component observed in Figure 5Aii remained on the dried PMEA surface (Figure 5Bi). Their size was approximately 50 nm in the lateral direction and 5 nm in height (Figure 5C), which is similar to the size of the FNG as previous reported (Hall and Slayter, 1959; Hu et al., 2016). Therefore, these grains were attributed to the FNG adsorbed onto the PMEA interface. In Figure 5Aiii, the locations of the adsorbed FNG molecules in the phase image (dark granular components) are shown in red on the topographic image. We noticed that the FNG molecules are mainly located in Domain A, or at their edges, rather than in Domain B. Furthermore, the amount of adsorbed FNG in the scanned area clearly decreased on the rinsed and dried PMEA surface (Figures 5Bi–iii). This substantiates a previous report using QCM (Tanaka et al., 2002), which demonstrated that FNG molecules adsorbed on a PMEA surface easily detach owing to the low denaturation at the interface. Therefore, we found through AFM observation that the FNG at Domain A, or the boundary, is in a nearly natural state and is easily detached. FNG molecules on the surface of the PBA could not be clearly distinguished using AFM, perhaps because a large number of FNG molecules were adsorbed on the entire surface.

## Conclusion

The amount of adsorbed fibrinogen and exposure of the γ chain on a PMEA surface were determined to be extremely lower than those on PBA, as shown through a microBCA and ELISA. Interaction measurements between the polymers and FNG at a microscopic scale using AFM revealed that the polymer-rich domains of PMEA and PBA, and the water-rich domains of PBA, showed an attraction force with FNG. In contrast, the water-rich domains of the PMEA showed no attraction and only repulsion. In accordance with the above results, the adsorbed FNG on the PMEA interface in the FNG solution was distributed in the polymer-rich domains, not in the water-rich domains. Furthermore, the amount of adsorbed FNG evidently decreased when rinsed with water, demonstrating that the FNG on the surface of the PMEA tends to easily desorb even in the polymer-rich domains.

Thus, the effect of the interfacial nano-structures generated through a phase separation at the interface on the protein adsorption behavior was quantitatively demonstrated in this study. We expect that the different FNG adsorption behaviors in the domains of the PMEA and PBA occur from the difference in polymer density and hydration structure. A further investigation of the relationship between the polymer density and hydration structure, and the protein adsorption and cell adhesion, are now ongoing and will be reported in a future study.

## Author contributions

TU carried out all experiments and wrote the manuscript. DM and MT directed and supervised the work.

### Conflict of interest statement

The authors declare that the research was conducted in the absence of any commercial or financial relationships that could be construed as a potential conflict of interest.
