# Supplementary material for: Analysis of Interaction Between Interfacial Structure and Fibrinogen at Blood-Compatible Polymer/Water Interface
Source: Front Chem. 2018 Nov 8;6:542. doi: 10.3389/fchem.2018.00542 (PMC6236912; doi:10.3389/fchem.2018.00542)
Supplement: Supplementary file 1 [file Data_Sheet_1.PDF]

Supplementary Material

Analysis of interaction between interfacial structure and fibrinogen at blood-compatible polymer/water interfaces

Tomoya Ueda[1], Daiki Murakami[1,2*], Masaru Tanaka[1,2,3*]

[1]Graduate School of Engineering, Kyushu University, Japan.

[2] Institute for Materials Chemistry and Engineering, Kyushu University, Japan.

[3]Frontier Center for Organic System Innovations, Yamagata University, Japan.

*** Correspondence:**

Masaru Tanaka: masaru_tanaka@ms.ifoc.kyushu-u.ac.jp

Daiki Murakami: daiki_murakami@ms.ifoc.kyushu-u.ac.jp

Supplementary Table 1. The static contact angle of sessile water droplet and captive air bubble on the polymer samples coated on polyethylene terephthalate (PET) substrates. The contact angles were measure at 30 seconds after deposition of water droplet or air bubble. These data represent means ± SD (*n* = 5 points on 3 samples for each polymer).

polymer	contact angle (deg)	
	sessile water drop	captive air bubble
PET	78.9 (±0.8)	121.7 (±0.8)
PET (coating surface)	75.0 (±0.5)	129.8 (±1.6)
PMEA	45.4 (±2.1)	133.7 (±2.4)
PBA	87.6 (±3.9)	126.9 (±1.6)

Supplementary Figure 1. Chemical structures of (A) poly(2-methoxyethyl acrylate) (PMEA) and (B) poly(butyl acrylate) (PBA).

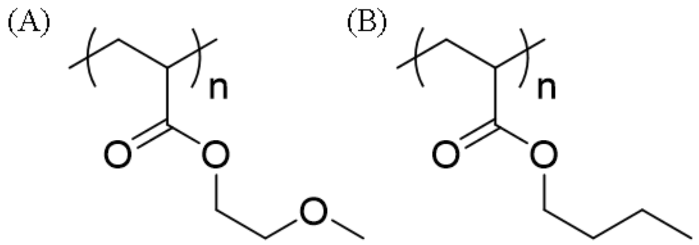

Supplementary Figure 2. AFM images of (A) PET / phosphate-buffered saline (PBS) interface, (B) PMEA surface in air, (C) PMEA / PBS interface, and (D) PBA / PBS interface. Scale bar is 1000 nm. (i) Topographic images, (ii) 3D images, (iii) phase images.

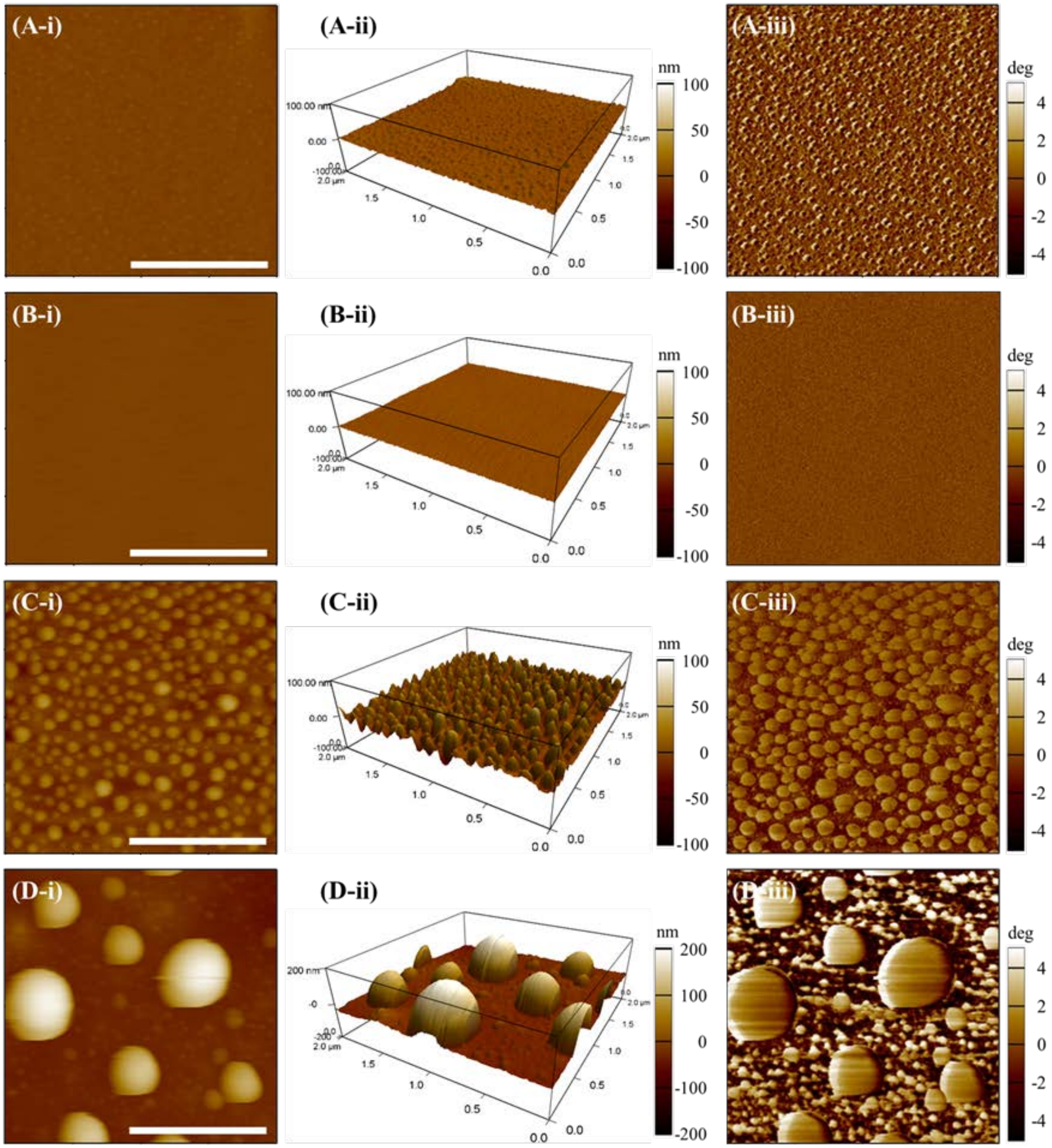

Determination of the detection limit

The detection limit in force measurement by AFM is basically calculated by following equation (Tseng, 2011).

$$\Delta F_{min} = \sqrt{k_{\mathrm{B}} T k_c}$$

Here, ΔF_{min} is detection limit, k_{B} is Boltzman constant, T is temperature, k_c is spring constant. In the present work, $\Delta F_{min} \approx 57$ pN. However, some extra factors like the effect of medium and shape of cantilevers affect the determination of the detection limit in actual case. In this work, we determined the detection limit of the force measurement from the fluctuation of base line signal. In Supplementary Figure 3, the magnified view of the base line obtained in our measurement is shown. The base line fluctuated within the force value of 30 pN. So we determined the detection limit in this work as 30 pN, and the forces detected over 30 pN were regarded as substantial repulsive or attractive interactions.

Supplementary Figure 3. Force curve measurement in approaching process with the combination of the PMEA and fibrinogen coated tip in PBS. (A) polymer-rich domain, (B) water-rich domain. (i) Force curve profile, (ii) magnified view of base line surrounded by a red frame in (i), (iii) magnified view of area surrounded by a blue frame in (i).

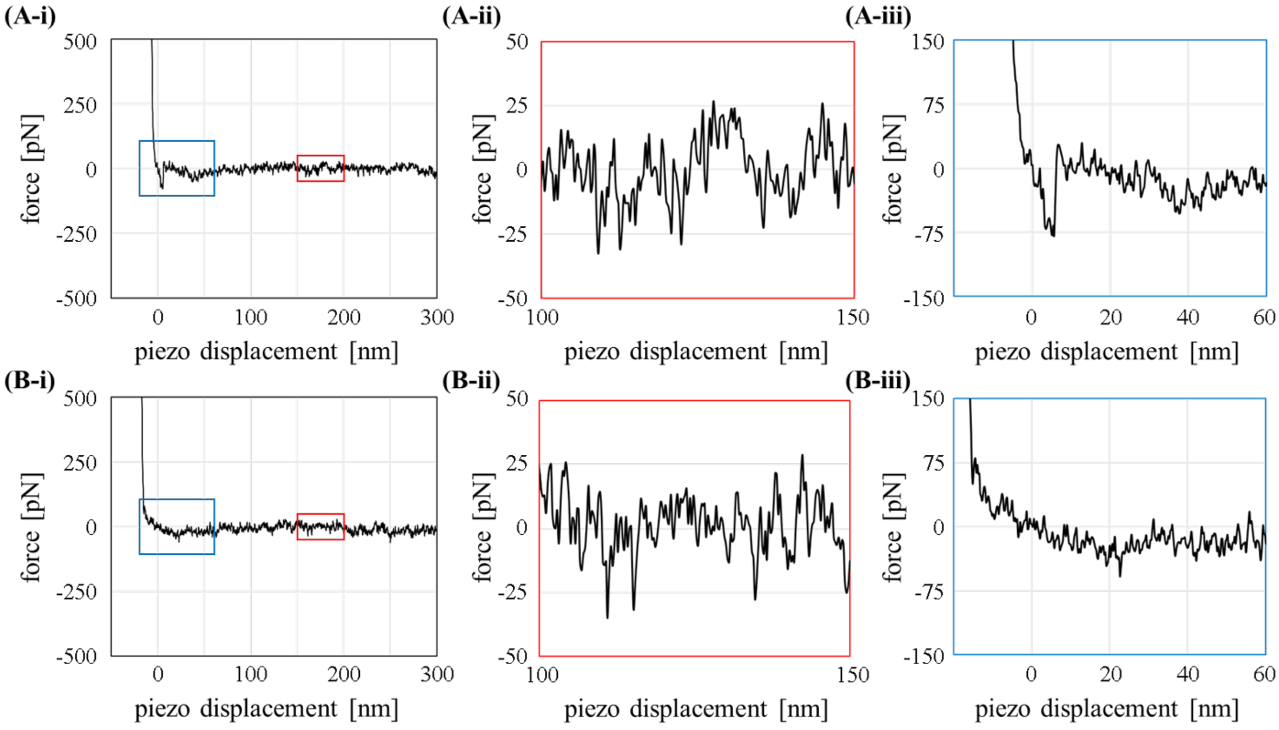

Supplementary Figure 4. Force curve measurement on approaching process with the combination of the PET and fibrinogen coated tip in PBS. (A) Force curve profiles, (B) The histogram of adhesion force at different 64 points.

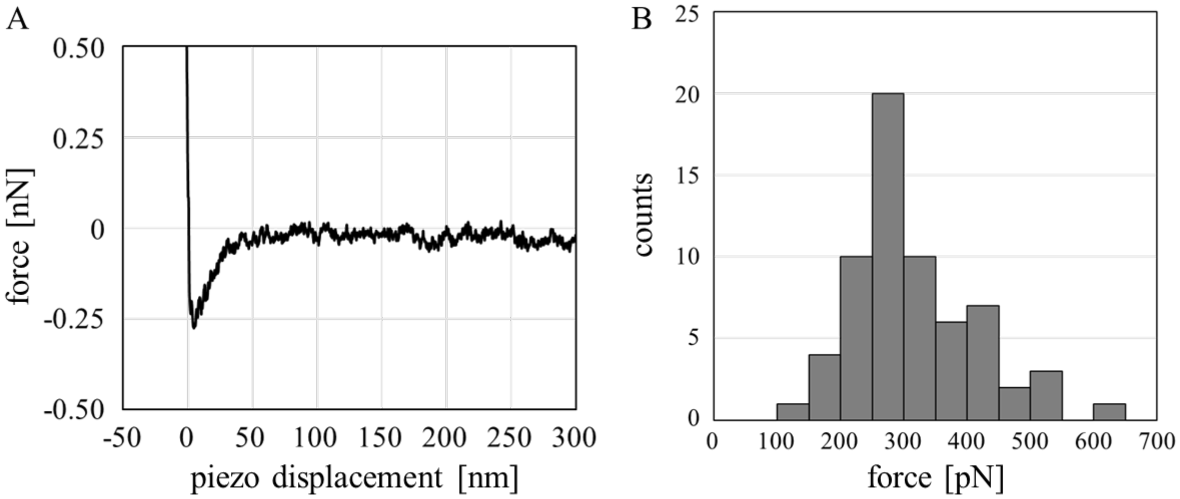

Reference

Tseng, A. A. (2011). *Tip-based nanofabrication : fundamentals and applications*. Springer.
